# A Viral-Vectored Multi-Stage Malaria Vaccine Regimen With Protective and Transmission-Blocking Efficacies

**DOI:** 10.3389/fimmu.2019.02412

**Published:** 2019-10-15

**Authors:** Yenni Yusuf, Tatsuya Yoshii, Mitsuhiro Iyori, Hiroaki Mizukami, Shinya Fukumoto, Daisuke S. Yamamoto, Talha Bin Emran, Fitri Amelia, Ashekul Islam, Intan Syafira, Shigeto Yoshida

**Affiliations:** ^1^Laboratory of Vaccinology and Applied Immunology, Kanazawa University School of Pharmacy, Kanazawa University, Kanazawa, Japan; ^2^Department of Parasitology, Faculty of Medicine, University of Hasanuddin, Makassar, Indonesia; ^3^Division of Genetics Therapeutics, Centre for Molecular Medicine, Jichi Medical University, Shimotsuke, Japan; ^4^National Research Centre for Protozoan Diseases, Obihiro University of Agriculture and Veterinary Medicine, Obihiro, Japan; ^5^Division of Medical Zoology, Department of Infection and Immunity, Jichi Medical University, Shimotsuke, Japan

**Keywords:** multi-stage malaria vaccine, transmission-blocking, *Plasmodium falciparum* circumsporozoite protein, Pfs25, human adenovirus serotype 5, adeno-associated virus

## Abstract

Malaria parasites undergo several stages in their complex lifecycle. To achieve reductions in both the individual disease burden and malaria transmission within communities, a multi-stage malaria vaccine with high effectiveness and durability is a more efficacious strategy compared with a single-stage vaccine. Here, we generated viral-vectored vaccines based on human adenovirus type 5 (AdHu5) and adeno-associated virus serotype 1 (AAV1) expressing a fusion protein of the pre-erythrocytic stage *Plasmodium falciparum* circumsporozoite protein (PfCSP) and the transmission-blocking sexual stage P25 protein (Pfs25). A two-dose heterologous AdHu5-prime/AAV1-boost immunization regimen proved to be highly effective for both full protection and transmission-blocking activity against transgenic *P. berghei* parasites expressing the corresponding *P. falciparum* antigens in mice. Remarkably, the immunization regimen induced antibody responses to both PfCSP and Pfs25 for over 9 months after the boosting and also maintained high levels of transmission-reducing activity (TRA: >99%) during that period, as evaluated by a direct feeding assay. If similar efficacies on *P. falciparum* can be shown following vaccination of humans, we propose that this multi-stage malaria vaccine regimen will be a powerful tool for malaria control, providing greater overall protection and cost-effectiveness than single-stage vaccines.

## Introduction

Malaria in humans is caused by *Plasmodium* spp. parasites, which undergo a complex lifecycle (1). The parasites are transmitted by *Anopheles* mosquitoes via the injection, during a bloodmeal, of sporozoites into the subcutaneous tissue, from which the sporozoites migrate to the liver and invade hepatocytes, then mature into schizont-containing merozoites that subsequently invade red blood cells, commencing the erythrocytic cycle. Some merozoites differentiate into gametocytes, which can be ingested by female mosquitoes, inside which they recombine into ookinetes that develop into oocysts in the mosquito midgut. The oocysts contain many sporozoites that migrate to the salivary gland and repeat the cycle in the next host. Throughout their lifecycle, the antigenic characteristics of the parasites change, the majority are not expressed at all stages; consequently, malaria vaccines specifically target different stages: pre-erythrocytic stage, erythrocytic stage, and sexual stage (2). The pre-erythrocytic stage is a prime target for intervention efforts because immunity against this stage is sterilizing; it prevents sporozoites from invading hepatocytes and/or it inhibits the development of liver-stage parasites into merozoites, thus eventually precluding the development of disease and the transmission of malaria (2, 3). This stage is the target of the RTS,S/AS01 vaccine, currently the most advanced malaria vaccine candidate, which acts through the induction of high levels of both anti-circumsporozoite (CSP) antibodies and CSP-specific CD4^+^ T cells, with a greater role being attributed to the antibody (4). However, in recent years, there has been an increased focus on the development of vaccines capable of breaking the cycle of *Plasmodium* by targeting the parasite sexual stages [transmission-blocking vaccines (TBV)], some of which have entered clinical trials (5–7). Both vaccine types, the pre-erythrocytic vaccines (PEV), and TBV, are categorized as vaccines that interrupt malaria transmission (VIMT) to support malaria elimination (8). Malaria vaccines are considered amongst the most important modalities for potential disease prevention and transmission reduction.

In 2013, the World Health Organization (WHO) malaria vaccine roadmap set two strategic goals to be met by 2030: (1) the development of vaccines that are highly efficacious in preventing clinical malaria and (2) the development of vaccines that prevent transmission, to accelerate malaria parasite elimination (7). An efficacious vaccine must either be completely effective against a stage, by eliminating over time the parasite or dramatically reducing parasite numbers, or else target multiple stages of the parasite lifecycle (2). Since such an effective vaccine is not yet available, the combination of partially effective vaccines that target different parasite stages provides another powerful way to achieve the WHO goals. A recent study demonstrated that partially efficacious interventions separately targeting the pre-erythrocytic and sexual stages have a synergistic impact in eliminating malaria from a population over multiple generations (9). Several studies have investigated the application of a mixture or co-administration of vaccines targeting different stages (10–14), including the most recent combination of RTS,S/AS01 and the current leading TBV candidate Pfs25-IMX313 (15), with some promising results.

Notably, a multi-stage vaccine, a vaccine targeting different stages of parasite life cycle in one construct, may provide a more cost-effective solution than a vaccination approach that uses mixtures of multiple single-stage vaccines. In addition, this approach may also be more convenient for the vaccine recipients than the co-administration of multiple vaccines. Unfortunately, there has not been much success yet in the development of such a multi-stage malaria vaccine (5). Several studies investigating potential multi-stage malaria vaccines found generally poor antibody responses and limited efficacies (16–19).

The progress of viral-vectored vaccines for malaria through the clinical development pathway has accelerated considerably (20). Very recently, our group generated a series of human adenovirus 5 (AdHu5) and adeno-associated virus serotype 1 (AAV1) expressing either *P. falciparum* pre-erythrocytic PfCSP or sexual stage Pfs25 antigen. Heterologous two-dose immunization with an AdHu5-prime and AAV1-boost elicited a high level of protection against sporozoite challenge and excellent TB activity with sustained high-titer antibody responses (21). Using the same platform, the present study aimed to develop a potent multi-stage malaria vaccine to effectively induce durable immunity for both protection and TB, which lasts for at least one transmission season (~6 months). In pursuit of this aim, we generated AdHu5- and AAV1-vectored multi-stage vaccines harboring “the *pfs25*-*pfcsp* fusion gene.” Transgenic *P. berghei* expressing either the *pfcsp* or *pfs25* genes were used to evaluate the protective and TB efficacies of this candidate multi-stage vaccine in a murine model. Our results demonstrate that the multi-stage vaccine regimen has the potential to fulfill the landmark goals of the malaria vaccine technology roadmap by achieving sterile protection and long-term TB efficacy.

## Materials and Methods

### Ethics Statement

All animal care and handling procedures were approved by the Animal Care and Use Committee of Kanazawa University (No. AP-163700) and in accordance with the Guidelines for Animal Care and Use prepared by Jichi Medical University (No. 09193). All efforts were made to minimize suffering in the animals.

### Parasites and Animals

The transgenic *P. berghei* Pfs25DR3 (*Pb*Pfs25DR3) that was used for TB assays and the ookinete immunofluorescence assays (IFAs) was kindly donated by A. Blagborough from Imperial College London (22). The transgenic *P. berghei* expressing PfCSP (PfCSP-Tc/Pb) that was used for the IFAs and the protective efficacy study was described previously (21–23). Both transgenic parasites were maintained in the Laboratory of Vaccinology and Applied Immunology, Kanazawa University. *Anopheles stephensi* mosquitoes (SDA 500 strain) were infected with the transgenic parasites by allowing them to feed on parasite-infected 6-week-old ddY mice. All other animal experiments used 6-week-old BALB/c mice.

### Viral Vector Construction

For the generation of AdHu5-Pfs25-PfCSP, the gene encoding Pfs25 and the Gly6Ser hinge was excised from pUC57-Simple-sPfs25-hinge (21) by digestion with EcoRI/MefI and then inserted into the EcoRI site of pENTR-D-sPfCSP2-G2-sWPRE (23) to construct pENTR-D-fusion. Pfs25-PfCSP was excised by digestion of pENTR-D-sPfs25-sPfCSP-WPRE with EcoRI/XmaI and then inserted into the EcoRI/XmaI sites of pENTR-CAG-sPfCSP2-G2-sWPRE (21) to construct pENTR-CAG-sPfs25-sPfCSP2-G2-sWPRE, which was subsequently cloned into the shuttle vector pAd/PL-DEST (Invitrogen, Carlsbad, CA, USA) using a Gateway cloning technology. The adenovirus was purified and titrated as described previously (24). For the generation of AAV1-Pfs25-PfCSP, the gene cassette encoding the fusion Pfs25-PfCSP was excised from pENTR-CAG-sPfs25-sPfCSP2-G2-sWPRE by digestion with KpnI and XhoI and then inserted into the KpnI and XhoI sites of pAAV-CMV-sPfs25 (21). The resulting plasmid, pAAV-CMV-sPfs25-sPfCSP2, was used to generate AAV1-Pfs25-PfCSP in HEK293 cells as described previously (25).

### Immunoblotting

HEK293 cells were infected with the AdHu5 vaccine at a Multiplicity Of Infection (MOI) of 10 or with the AAV1 vaccine at a MOI of 10^6^. Cell lysates were collected using Laemmli buffer at 48 h post-infection and subjected to immunoblotting as described previously (24). The cell lysates were electrophoresed on 10% sodium dodecyl sulfate polyacrylamide (SDS-PAGE) gels under reducing conditions for probing with the anti-PfCSP monoclonal antibody (mAb) 2A10 or under non-reducing conditions for probing with anti-Pfs25 mAb 4B7. Each blot was visualized using an Odyssey infrared imager (LI-COR, Lincoln, NE, USA).

### Immunofluorescence Assay

IFAs to assess protein expression were performed as described previously (21). HEK293 cells were infected with the AdHu5 vaccine or AAV1 vaccine on an eight-well-chamber slide at a MOI of 10 or 10^5^, respectively. Cells were fixed with 100% methanol (permeabilized) or 4% paraformaldehyde (non-permeabilized) for 30 min at 24 h post-infection. To visualize the expression of the antigen, the cells were incubated for 1 h at room temperature with Alexa-Fluor-488-conjugated 2A10 and Alexa-Fluor-596-conjugated 4B7, each diluted 1:100.

For IFAs of sporozoites and ookinetes, the sporozoites were isolated from mosquito salivary glands and then fixed with acetone/methanol (1:1) on glass slides, while the ookinetes were isolated from infected blood culture and then fixed with 4% paraformaldehyde. After blocking with 10% normal goat serum, slides were incubated with sera from immunized mice (1:80 dilution) for 1 h, followed by incubation with FITC-conjugated goat anti-mouse IgG for 1 h. For positive controls, sporozoites and ookinetes were stained with Alexa-Fluor 596-conjugated 2A10 and 4B7, respectively. In all IFA experiments, VECTASHIELD® containing 4′, 6-diamidino-2-phenylindole (DAPI) was used for nuclear staining, and a BZ-X710 fluorescence microscope (Keyence Corp, Tokyo, Japan) was used for image acquisition.

### Immunization

All vaccines were administered intramuscularly in 100 μl of phosphate-buffered saline (PBS). AdHu5 vaccines were administered at a dose of 5 × 107 plaque-forming units (PFU) as a prime, whereas AAV1 vaccines were administered at a dose of 1,011 vg per mouse as a boost. Immunization was performed with a 6-week interval between the prime and boost. For the vaccine mixture regimen, the same doses of AdHu5-PfCSP and AdHu5-Pfs25 (5 × 107 PFU) were mixed in a syringe as a prime, and the same doses of AAV1-PfCSP and AAV1-Pfs25 (1,011 vg) were mixed as a boost. Negative control animals were injected with either PBS or luciferase-expressing viruses (AdHu5-Luc-prime/AAV1-Luc-boost).

### ELISA

PfCSP- or Pfs25-specific antibody (Ab) levels were quantified by ELISA as described previously (21, 24). The PfCSP antigen was produced using an *Escherichia coli* expression system. The Pfs25 antigen was produced using a wheat germ cell-free (WGCF) protein expression system (CellFree Sciences, Matsuyama, Japan) (26). Sera from immunized mice were collected from tail vein blood samples 1 day before boost and 1 day before challenge, or weekly up to 280 days post-boost for monitoring. Pre-coated Costar®EIA/RIA polystyrene plates (Corning Inc., NY, USA) with 400 ng/well of PfCSP or 200 ng/well of Pfs25 were blocked with 1% bovine serum albumin (BSA) in PBS and then incubated with serially diluted sera samples, as well as with negative and positive controls (mAb 2A10 or mAb 4B7, respectively). An anti-mouse IgG conjugated with horseradish peroxidase (HRP) (Bio-Rad lab, Inc., Tokyo, Japan) was used as the secondary Ab. The endpoint titer is expressed as the reciprocal of the last dilution that gave an optical density at 414 nm of 0.15 U above the values of the negative controls (<0.1). All mice used in our experiments were seronegative before immunization.

### Parasite Challenge Test

Mice were intravenously challenged with PfCSP-Tc/Pb sporozoites resuspended in RPMI 1640 media (Gibco, Life Technologies, Tokyo, Japan). Sporozoites were prepared as described previously (27). Each mouse was injected via the tail vein with 100 μl of media containing 500 sporozoites. Infection was monitored from day 4 to 14 by Giemsa staining of thin blood smears obtained from the tail. Protection was defined as the complete absence of blood-stage parasitemia on day 14 post-challenge. The time required to reach 1% parasitemia was determined as described previously (28).

### TB Assays

TB was assessed using direct feeding assays (DFAs) as described previously (21). At 35 or 287 days after boost, mice were treated with phenylhydrazine and then infected intraperitoneally with 10^6^ *Pb*Pfs25DR3-parasitized red blood cells (pRBCs) 3 days later. At 3 days post-infection, at least 50 starved *A. stephensi* mosquitoes were allowed to feed on each infected mouse. At 5–6 h post-feeding, any unfed mosquitoes were removed. Mosquitoes were then maintained on fructose [8% (w/v) fructose, 0.05% (w/v) *p*-aminobenzoic acid] at 19–22°C and 50–80% relative humidity. On day 10–12 post-feeding, the mosquito midguts were dissected, and oocyst prevalence and intensity were recorded. For each mouse, the number of oocysts was counted, and the mean oocyst intensity was calculated. For inhibition calculations, these numbers were compared with those of mice immunized with the AdHu5-AAV1 Luc control. Percent (%) inhibition of mean oocyst intensity (transmission-reducing activity; TRA) was calculated as follows: 100 × [1 – (mean number of oocysts in the test group/mean number of oocysts in the control groups)]. Similarly, the % inhibition of oocyst prevalence (transmission-blocking activity; TBA) was evaluated as: 100 × [1 – (proportion of mosquitoes with any oocysts in the test group/proportion of mosquitoes with any oocysts in the control groups)] (29).

### Statistical Analysis

For all statistical analyses, GraphPad Prism version 7.0 for Mac OS was used. Depending on the data distribution, a Student's *t*-test or Mann-Whitney rank test was used for comparing two groups. All ELISA end-point titers were log_10_-transformed before analysis. The proportion of mice not reaching 1% parasitemia was analyzed using a Kaplan-Meier log-rank (Mantel-Cox) test. The significance of TRA and TBA was assessed using the Mann-Whitney *U*-test and Fisher's exact probability test, respectively. A *p* < 0.05 was considered statistically significant.

## Results

### Both AdHu5 and AAV1 Vaccines Expressed the Pfs25-PfCSP Fusion Antigen on the Surface of Mammalian Cells *in vitro*

**Figure 1:**
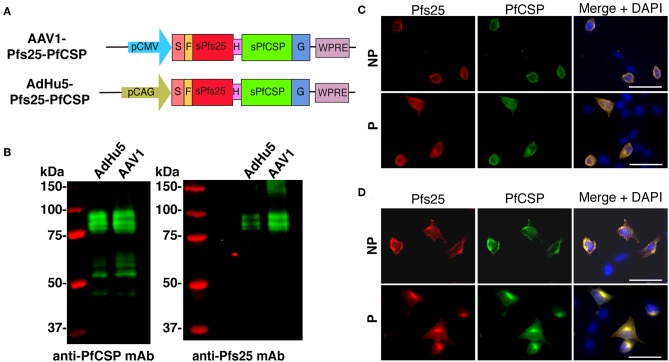
Construction of viral-vectored vaccines. **(A)** Expression of the *pfcsp* and *pfs25* gene cassettes was driven by the CMV promoter in AAV1 and by the CAG promoter in AdHu5. S, signal sequence; F, FLAG epitope tag; H, Hinge G6S; G, VSV-G transmembrane protein. **(B)** Analysis of the PfCSP and Pfs25 expression in HEK293T cells transduced with AdHu5-Pfs25-PfCSP (MOI = 10) or AAV1-Pfs25-PfCSP (MOI = 10^6^). Cells were lysed and loaded onto a 10% SDS-PAGE gel and immunoblotted with anti-PfCSP mAb 2A10 and anti-Pfs25 mAb 4B7. **(C,D)** Localization of PfCSP and Pfs25 expression in mammalian cells after transduction with AdHu5-Pfs25-PfCSP **(C)** or with AAV1-Pfs25-PfCSP **(D)**. After 24 h, the cells were fixed with paraformaldehyde (NP; non-permeabilized) or methanol (P; permeabilized) and blocked with 10% normal goat serum. After being blocked, the cells were incubated with Alexa-Fluor-488-conjugated anti-PfCSP mAb (green) and Alexa-Fluor-594-conjugated anti-Pfs25 mAb (red). Cell nuclei were visualized with DAPI (blue). Original magnification, 400×. Scale bars = 50 μm.

The *pfs25*-*pfcsp* fusion gene was linked by a sequence encoding a flexible hinge peptide (Gly_6_Ser) between the *pfcsp* and *pfs25* genes, and its expression was driven by the CAG and CMV promoter in AdHu5 and AAV1, respectively (Figure 1A). In the previous study, we reported that Pfs25 reacted with mAb 4B7 in a ladder of bands with relative *Mr* of 33–48 kDa due to post-translational modifications because there are two potential *N*-linked glycosylation sites in the predicted amino acid sequence of Pfs25 (21). Similarly, the present study revealed that the Pfs25-PfCSP fusion protein in the cells infected with AdHu5 (MOI = 10, lane 1) or AAV1 (MOI = 10^6^, lane 2) reacted with both mAb 2A10 (anti-PfCSP) and 4B7 (anti-Pfs25) in a ladder of bands with relative *Mr* of 80–100 kDa (Figure 1B). In addition, there were several cleaves of the fusion protein causing the presence of a single band of PfCSP with relative *Mr* of 53 kDa in the blot of protein with anti-PfCSP (Figure 1B, left panel). An IFA analysis demonstrated that both Pfs25 and PfCSP epitopes were expressed both in the cytoplasm and on the surface of infected cells (Figures 1C,D). Since the mAb 4B7 recognizes a conformation-dependent epitope of Pfs25, these results suggest that the Pfs25-PfCSP fusion antigen on the surface of the infected cells retain the three-dimensional structure of the native Pfs25 protein, which is essential for the induction of antibodies with TB functionality (30).

### The AdHu5-AAV1 Pfs25-PfCSP Regimen Induces Potent and Durable Anti-PfCSP and Anti-Pfs25 Immune Responses

**Figure 2:**
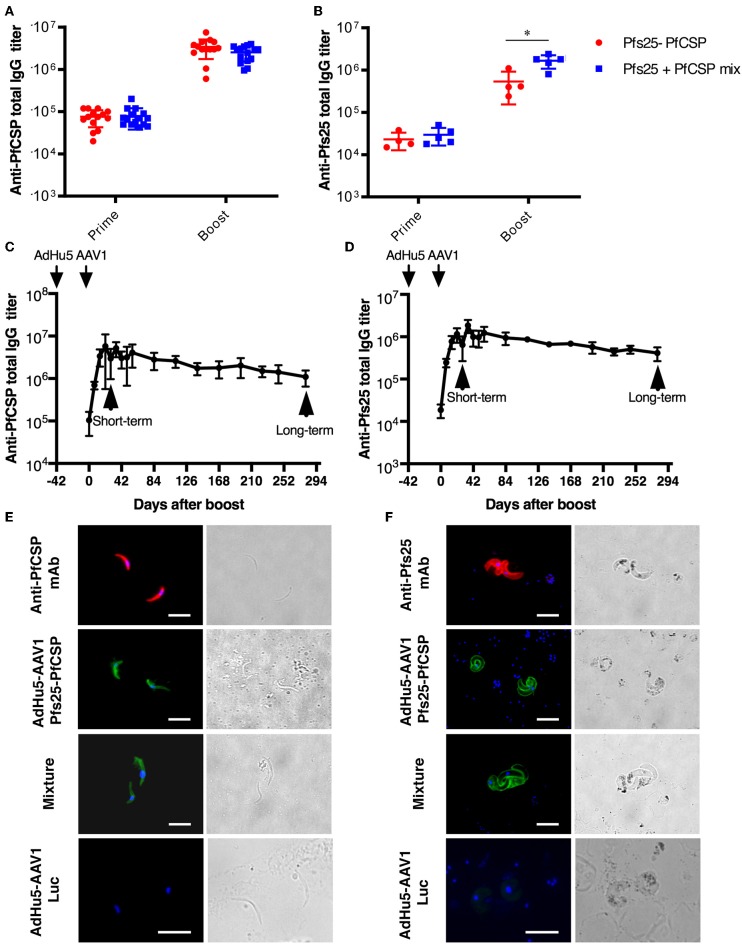
Immunogenicity of the AdHu5-AAV1 Pfs25-PfCSP. **(A,B)** Comparison of antibody responses induced by the multi-antigen vaccine and the mixture of single-antigen vaccines. BALB/c mice were immunized with the indicated regimen. Individual sera were collected 4 weeks after boost, and antibody titers against PfCSP **(A)** and Pfs25 **(B)** were measured using ELISAs. The AdHu5-AAV1 Pfs25-PfCSP are labeled as “Pfs25-PfCSP”; the data from priming with a mixture of AdHu5-Pfs25 and AdHu5-PfCSP and boosting with a mixture of AAV1-Pfs25 and AAV1-PfCSP are labeled as “Pfs25+PfCSP mix.” Each datapoint represents a single mouse. Horizontal lines indicate the means of antibody titers [± standard deviation (SD)]. Differences between groups were assessed with a Mann-Whitney *U*-test; ^*^p < 0.05. **(C,D)** Monitoring of antibody responses. BALB/c mice (*n* = 5–10) were immunized with the AdHu5-AAV1 Pfs25-PfCSP at 6-week interval. Individual sera were collected 1 day before the boost, then weekly after the boost up to 280 days. Antibody titers against PfCSP **(C)** and Pfs25 **(D)** were measured using ELISAs. Data are presented as the mean ± SD. **(E,F)** Reactivity of immune sera with the transgenic parasites. The transgenic PfCSP-Tc/Pb sporozoites **(E)** and ookinetes of Pfs25DR3 Pb **(F)** were fixed and incubated with sera from the immunized mice described in **(A,B)** and stained with FITC-conjugated goat anti-mouse IgG (green) for IFAs. The sporozoites and ookinetes were incubated with Alexa-594-conjugated 2A10 and 4B7, respectively, as positive controls. Cell nuclei were visualized with DAPI (blue). Original magnification, 1,000×. Scale bars = 10 μm.

To investigate the immunogenicity of the AdHu5-Pfs25-PfCSP-prime/AAV1-Pfs25-PfCSP-boost heterologous regimen (AdHu5-AAV1 Pfs25-PfCSP), first we determined the PfCSP- and Pfs25-specific antibody responses induced by the fusion vaccines as compared with those induced by the mixture of corresponding single-antigen vaccines. Mice were immunized with a 6-week interval between the prime and boost (*n* = 15). Our preliminary studies revealed that mice immunized with the adenovirus-prime/AAV-boost regimen achieved peak antibody titer at 4 weeks after boost. Therefore, at 4 weeks post-boost, sera of the immunized mice were collected for ELISA (*n* = 14–15 for PfCSP analysis; *n* = 4–5 for Pfs25 analysis). Both anti-PfCSP and anti-Pfs25 IgG titers were induced by the AdHu5-AAV1 Pfs25-PfCSP. Although the anti-Pfs25 IgG titer in the fusion group was slightly lower after boost compared with that in the mixture group (537,500 vs. 1,660,000) (Figure 2, the anti-PfCSP IgG titers were comparable between both groups (3,439,286 vs. 2,536,667) (Figure 2A). Antibody monitoring of the animals immunized with the fusion vaccines showed that the IgG titers against both PfCSP and Pfs25 remained high after 280 days (Figures 2C,D). The IFA results demonstrated that immune sera from mice vaccinated with the fusion vaccines reacted with the transgenic sporozoites (Figure 2E) and ookinetes (Figure 2F), at levels comparable to those observed for the immune sera from mice vaccinated with the mixture of single-antigen vaccines.

### The AdHu5-AAV1 Pfs25-PfCSP Confers Complete Protection Against Transgenic *P. berghei* Expressing PfCSP

**Figure 3:**
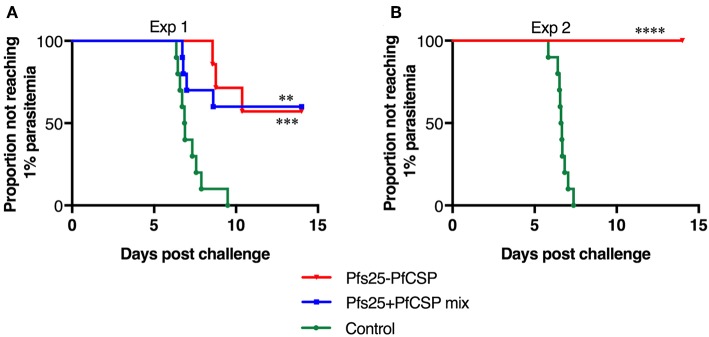
Protective efficacy of the AdHu5-AAV1 Pfs25-PfCSP. BALB/c mice (*n* = 10) were immunized with the indicated regimen at 6-week interval. Four weeks after boost, the mice were challenged with an intravenous injection of 500 transgenic PfCSP-Tc/Pb sporozoites. Parasitemia was monitored for 3 consecutive days, starting from day 4 after challenge, and a model predicting the time to reach 1% parasitemia was generated. The absence of blood-stage parasites in the animals was confirmed on day 14 after challenge. The AdHu5-AAV1 Pfs25-PfCSP are labeled as “Pfs25-PfCSP” **(A,B)**; data from mice primed with a mixture of AdHu5-Pfs25 and AdHu5-PfCSP and boosted with a mixture of AAV1-Pfs25 and AAV1-PfCSP are labeled as “Pfs25+PfCSP mix” **(A)**. The statistical analysis was performed by generating Kaplan-Meier survival curves, and *p-*values were calculated with a Kaplan-Meier log-rank (Mantel-Cox) test. ^****^p < 0.0001, ^***^p < 0.001, and ^**^p < 0.01 as compared with control groups.

To assess the protective efficacy of the AdHu5-AAV1 Pfs25-PfCSP, immunized mice were challenged with PfCSP-Tc/Pb sporozoites at 4 weeks post-boost, and the presence of blood infection was evaluated up to 14 days post-challenge. For comparison, the mice immunized with the mixture regimen were challenged at the same time. The fusion vaccine regimen conferred 57% protection, which is comparable with that of the mixture regimen (60%) (Figure 3A). In another challenge experiment evaluating protective efficacy of the fusion vaccine only, the protection level reached 100% (Figure 3B). Since the difference of mean antibody titer between both fusion groups was not statistically significant (3,365,000 vs. 3,775,000), we assumed that the variability in the protection level between the two experiments was due to the high variability of the intravenous sporozoite challenge itself as described by Leitner et al. (31).

### The AdHu5-AAV1 Pfs25-PfCSP Elicits a Durable TB Efficacy, Lasting at Least 287 Days

**Figure 4:**
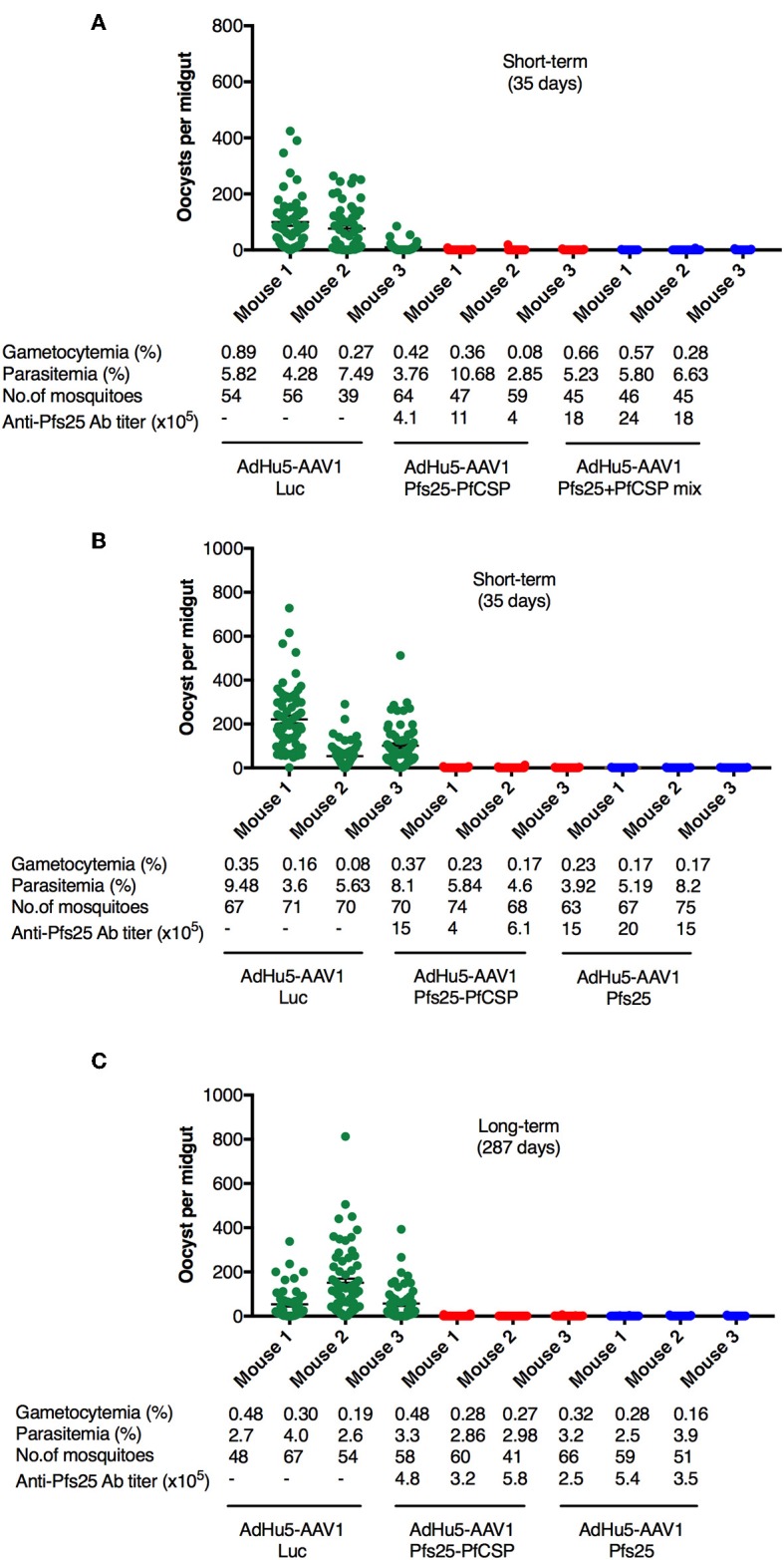
TB efficacy of various immunization regimens. BALB/c mice were immunized with the indicated regimen at 6-week interval (*n* = 3), then infected with *Pb*Pfs25DR3 at 35 days **(A,B)** or 287 days **(C)** after boost. Mosquitoes were allowed to feed on the infected mice in a DFA. At day 10–12 post-feeding, the mosquito midguts were dissected, and oocyst intensity and prevalence were determined [Table 1, Exp.1 **(A)**, Exp.2 **(B)**, and Exp.3**(C)**]. Each datapoint represents a single blood-fed mosquito. The x-axis columns represent individual mice. Horizontal lines indicate the mean numbers of oocysts observed [± standard errors of the means (SEM)].

It is widely accepted that the transmission blocking efficacy relates directly to the anti-Pfs25 Ab titer (32); thus, we expected the AdHu5-AAV1 Pfs25-PfCSP to have a high TB efficacy. To evaluate the functional activity of the anti-Pfs25 Ab induced by AdHu5-AAV1 Pfs25-PfCSP, we assessed the TB efficacies at 35 days (short-term) and 287 days (long-term) after boost by performing DFAs. Groups of five mice were infected intraperitoneally with 10^6^ *Pb*Pfs25DR3-pRBCs. At 3 days after infection, three of the five mice in each group were chosen for DFA based on their parasitemia (>2%) and gametocytemia (>0.05%) (Figure 4). *A. stephensi* mosquitoes were allowed to feed on each infected mouse, and the oocyst intensity and prevalence were recorded at 10–12 days post-feeding. Reductions in intensity and prevalence in the immunized mice were calculated with respect to the AdHu5-AAV1 Luc-immunized controls.

First, we evaluated the short-term TB efficacy of the fusion vaccines as compared with the mixture of single-antigen vaccines (Table 1, Exp.1, Figure 4A). In this experiment, mosquitoes that fed on the three control mice displayed an average intensity of 61.87 oocysts/midgut. Following AdHu5-AAV1 Pfs25-PfCSP immunization, the mean intensity was only 0.29 oocysts/midgut, corresponding to a reduction (referred to as the TRA) of 99.53% (*p* < 0.0001), compared with the mean intensity of 0.16 oocysts/midgut in the mixture group (TRA of 99.74%, *p* < 0.0001). Correspondingly, the mean infection prevalence was reduced from 83.59 to 6.94% and 5.91% in the AdHu5-AAV1 Pfs25-PfCSP group and the mixture group, respectively, achieving significant reductions (referred to as the TBA) of 91.70% (*p* < 0.0001) and 92.93% (*p* < 0.0001). These results demonstrate that although there was a slight difference in the anti-Pfs25 IgG titers between the fusion vaccine group and the mixture group, there were no significant differences in the TRA or TBA between these groups (*p* = 0.67 and *p* = 0.82, respectively).

**Table 1:** Transmission-blocking activity of the AdHu5-Pfs25-PfCSP prime/AAV1-Pfs25-PfCSP boost immunization regimen.

**Group**	**Prime**	**Boost**	**Mean intensity ± SEM** **(oocysts per midgut)**	**Mean prevalence ± SEM** **(% infected mosquitoes)**	**TRA (%)^a^^,^^b^**	**TBA (%)^c^^,^^d^**
**Exp.1. Short-term**						
Fusion	AdHu5-Pfs25-PCSP	AAV1-Pfs25-PfCSP	0.29 (0.07)	6.94 (2.47)	99.53^*^	91.70^*^
Mix	AdHu5-Pfs25 +AdHu5-PfCSP	AAV1-Pfs25 +AAV1-PfCSP	0.16 (0.05)	5.91 (1.98)	99.74^*^	92.93^*^
Control	AdHu5-Luc	AAV1-Luc	61.87 (26.92)	83.59 (11.21)		
**Exp.2. Short-term**						
Fusion	AdHu5-Pfs25-PCSP	AAV1-Pfs25-PfCSP	0.291 (0.04)	8.037 (1.00)	99.77^*^	91.73^*^
Pfs25 alone^e^	AdHu5-Pfs25	AAV1-Pfs25	0.20 ± 0.04	10.41 ± 3.51	99.84^*^	89.28^*^
Control	AdHu5-Luc	AAV1-Luc	125.2 (49.84)	97.18 (2.15)		
**Exp.3. Long-term**						
Fusion	AdHu5-Pfs25-PCSP	AAV1-Pfs25-PfCSP	0.42 (0.19)	16.44 (4.90)	99.52^*^	81.87^*^
Pfs25 alone^e^	AdHu5-Pfs25	AAV1-Pfs25	0.24 ± 0.03	12.72 ± 1.53	99.73^*^	85.97^*^
Control	AdHu5-Luc	AAV1-Luc	87.24 (31.86)	90.63 (5.67)		

a Transmission-reducing activity (TRA) was calculated by comparison with the control (AdHu5-AAV1 Luc) group, and significant differences were assessed using a Mann-Whitney U-test (^*^p < 0.0001).

b *No significant difference between the TRA of Pfs25-PfCSP and Pfs25+PfCSP mix (p = 0.6720) in Exp.1*.

c Transmission-blocking activity (TBA) was calculated by comparison with the control group, and significant differences were assessed using a Fisher's exact probability test (^*^p < 0.0001).

d *No significant difference between the TBA of Pfs25-PfCSP and Pfs25+PfCSP mix (p = 0.8171) in Exp.1*.

e *These results have been previously reported in (21)*.

Next, to re-confirm the TB efficacy of the AdHu5-AAV1 Pfs25-PfCSP, we performed another short-term study (Table 1, Exp.2, Figure 4B). In this experiment, mosquitoes that fed on the three control mice displayed an average intensity of 125.17 oocysts/midgut and an infection prevalence of 97.18%. The mean intensity was 0.29 oocysts/midgut in the immunized group, achieving a TRA of 99.77% (*p* < 0.0001), and the mean infection prevalence was 8.04%, achieving a TBA of 91.73% (*p* < 0.0001). These results are comparable with those of Exp.1. In addition, there are no significant differences with our previous data on the short-term TRA and TBA of the AdHu5-Pfs25-prime/AAV1-Pfs25-boost (AdHu5-AAV1 Pfs25) (21).

Finally, we evaluated the long-term TB efficacy of the AdHu5-AAV1 Pfs25-PfCSP at 287 days after the booster injection (Table 1, Exp.3, Figure 4C). The experiment revealed that the TRA did not significantly decline over 287 days. The mean oocyst intensity in the immunized group was 0.42 oocysts/midgut (compared with 87.24 oocysts/midgut in the control group), achieving a TRA of 99.52% (*p* < 0.0001), and the mean infection prevalence was 16.44% compared with the 90.63% of the control group, achieving a TBA of 81.87% (*p* < 0.0001). This result similarly showed no significant differences with our previous data on the long-term TRA and TBA of the AdHu5-AAV1 Pfs25 (21).

Collectively, our data demonstrate that the AdHu5-AAV1 Pfs25-PfCSP is an effective multi-stage malaria vaccine, capable of inducing a high level of PfCSP- and Pfs25-specific Ab immune responses and achieving a high level of protective immunity and long-term TB immunity against the malaria parasite.

## Discussion

Here, we demonstrated that an immunization regime using AdHu5- and AAV1-based multi-stage malaria vaccines harboring a gene encoding the pre-erythrocytic antigen PfCSP fused with the mosquito-stage antigen Pfs25 is protective and has durable TB efficacy. The AdHu5-AAV1 prime-boost approach evoked high levels of antibody titers against both antigens, which were sustained for at least one transmission season; this is one of the desired features of an ideal malaria vaccine (33). Remarkably, the administration of these vaccines in a prime-boost combination achieved both protection and TB immunity in a murine model, fulfilling the urgent need for an effective second-generation malaria vaccine that reduces transmission and incidence, rather than simply reducing the morbidity and mortality of the disease (7, 34). Moreover, it may help meet the strategic goals for 2030 set by the WHO malaria vaccine roadmap (7).

The Malaria Eradication Research Agenda Consultative Group on Vaccines (malERACGoV) (8) requires that a VIMT must primarily reduce malaria transmission. A highly effective PEV that prevents erythrocytic stage infection will obviously reduce transmission, but the current leading PEV candidate, RTS,S/AS01, confers only 36.3% protection (35, 36). However, it has been suggested that a successful TBV would ideally be combined with a vaccine that blocks sporozoite/liver stage development and/or asexual blood stage development (37). Hence, mixing a PEV and TBV together might provide a means of achieving an effective VIMT. In the present study, we selected the immunogen Pfs25 to exert a TB effect because it was demonstrated that Pfs25 may induce functional antibodies in humans that effectively inhibit malaria parasite development in mosquitoes (38, 39). We focused on a pre-erythrocytic antigen, rather than a blood-stage antigen, for use in conjunction with Pfs25 because this combination was suggested as likely to be the most efficient in reducing malaria prevalence; a malaria model analysis of pathogen virulence evolution predicted that PEVs select for lower parasite virulence, while blood-stage vaccines select for higher parasite virulence, so a combination of these may increase the population-level benefits of vaccination (40).

The use of a multi-stage vaccine regimen may increase the adherence to a malaria elimination vaccination program because people will be more likely to go get vaccinated if the shot also provides personal protection from the disease. A recent study investigated the potential of mixing the RTS,S/AS01 with Pfs25-IMX313/AS01 in one formulation or co-administering both vaccines (15), and it found that the combination of these two vaccines elicited antibody titers against both PfCSP and Pfs25 that were similar to those elicited by the corresponding single-antigen vaccines. Using *in vitro* assays, it was also shown that this combination of vaccines exhibited similar functional activity in TB and sporozoite inhibition. Using *in vivo* assays, our results from mice immunized with the mixture of single-antigen formulations are in line with those of this previous study, in contrast with the studies that found immune interference or reduced efficacies for combinations of several other malaria vaccines (11, 41, 42).

Notably, mixing two or more vaccines in a single formulation can result in a higher vaccination cost. Thus, the development of a multi-stage vaccine harboring different antigens from different parasite stages may be the best way of reducing the vaccination cost. To address this issue, we employed the multi-stage AdHu5-AAV1 Pfs25-PfCSP and demonstrated that this regimen has a similar efficacy as the mixture of corresponding single-antigen formulations, in terms of both protection and TB activity. Remarkably, the TB activity lasted for over 9 months, with the sustained high titer of antibodies against Pfs25 exceeding one transmission season. These results are in contrast with those from previous attempts at the development of multi-stage malaria vaccines, which generally demonstrated poor antibody responses (16–19). A similar conjugated vaccine with a successful induction of antibody responses against both CSP and Pfs25 has been reported by only one group, Kubler-Kielb et al.; they used protein adjuvanted with alum (43) and did not evaluate the protective efficacy of the vaccine. Additionally, their study administered the vaccines via three doses with 2-week intervals, whereas the number of doses in our study was reduced to only two separated by a longer interval.

One limitation of this study is that the long-term protective efficacy of the regimen has not been investigated. This feature, both on mice that have not been challenged before and on mice that were protected from a challenge, will be evaluated in our future work. However, even if the long-term protective efficacy is lower than the protective efficacy observed here, this regimen could still be a great tool for supporting the malaria elimination program because anti-sporozoite and anti-transmission interventions may act synergistically to accelerate malaria elimination efforts over multiple generations (9). Moreover, the additional TB immunity combined with the pre-erythrocytic immunity conferred by this multi-stage regimen is an improvement over the RTS,S/AS01 for a next-generation vaccine (44). Thus, we are currently planning further evaluation of this regimen in human trials.

## Data Availability Statement

The raw data supporting the conclusions of this manuscript will be made available by the authors, without undue reservation, to any qualified researcher.

## Ethics Statement

The animal study was reviewed and approved by Animal Care and Use Committee of Kanazawa University, Guidelines for Animal Care and Use Jichi Medical University. Written informed consent was obtained from the owners for the participation of their animals in this study.

## Author Contributions

YY, MI, and SY: study concept and design, and drafting the manuscript. YY, TY, MI, SF, DY, TE, FA, AI, IS, and SY: acquisition of data. YY, TY, MI, and SY: analyses and interpretation of data. MI and SY: critical revision of the manuscript for important intellectual content. YY and TY: statistical analyses. YY, TY, MI, HM, SF, DY, TE, FA, AI, IS, and SY: technical or material support. SY: study supervision.

### Conflict of Interest

SY, MI, and HM are named inventors on filed patents related to immunization with the AAV anti-malaria vaccines. These products have not been commercialized. The remaining authors declare that the research was conducted in the absence of any commercial or financial relationships that could be construed as a potential conflict of interest.
